# COVID-19: Lockdowns, Fatality Rates and GDP Growth: Evidence for the First Three Quarters of 2020

**DOI:** 10.1007/s10272-021-0948-y

**Published:** 2021-01-26

**Authors:** Michael König, Adalbert Winkler

**Affiliations:** International and Development Finance, Frankfurt School of Finance and Management, Adickesallee 32–34, 60322 Frankfurt a. M., Germany

## Abstract

The COVID-19 pandemic has triggered an unprecedented economic crisis. This article analyses the impact of mandatory social distancing imposed by lockdown policies and voluntary social distancing triggered by COVID-19 fatality rates on GDP growth in the first three quarters of 2020 for a sample of 42 countries. OLS and IV results indicate an important role for the fatality rate, while panel regressions show that lockdown stringency is the more important driver of growth. When including lagged variables, more restrictive measures lead to lower GDP growth in the same quarter but are associated with a positive, catching-up effect in the following quarter.

The coronavirus pandemic has triggered a massive health crisis across the globe. More than 1.8 million people have died with or from COVID-19 in 2020 and more than 81 million (around 1% of global world population) were infected (WHO, 2021). Despite massive policy support, the global economy recorded a severe recession in the second quarter of 2020 (IMF, 2020). After a period of recovery in the third quarter, the second wave of the pandemic, which started in the autumn of 2020, led to another decline in economic activity.

While the pandemic is a global one, countries have been affected differently by the virus and have responded with different policies (Brauner et al., 2020; Hale et al., 2020). As a result, growth developments have varied across countries as well. This paper, an update and extension of König and Winkler (2020b), answers the question of whether and to what extent growth developments over the first three quarters of 2020 reflect differences in the intensity with which governments enacted restrictions, i.e. the economic effects of *mandatory* social distancing, and differences in the fatality rate associated with the virus, i.e. the economic effects of *voluntary* social distancing triggered by the fatality rate.

**Michael König**, Frankfurt School of Finance & Management, FS UNEP Collaborating Centre for Climate & Sustainable Energy Finance, Germany.

The paper is motivated by the debate on the economic implications of government-imposed restrictions and lockdowns responding to rising infection rates. When confronted with the first wave of the pandemic, governments took different positions in this debate. Some countries imposed social distancing rather hesitantly, such as the UK or the US, and lightly, such as Sweden (Born et al., 2020; Krueger et al., 2020). Thus, they relied on voluntary social distancing as they feared that the costs of mandatory restrictions would be too high even if they reduced health risks. Other countries enacted strict lockdowns, either because they recorded quickly rising infection and fatality rates, as was the case in Italy and Spain, or because governments considered the degree of voluntary social distancing as insufficient to keep the pandemic under control, e.g. in Germany or Denmark (Brauner et al., 2020; Farboodi et al., 2020). Moreover, they aimed at limiting the economic damage of an unrestrained spread of the virus, i.e. the direct costs such as the loss of working time and the rise in medical costs (Gros, 2020) as well as the costs associated with voluntary social distancing triggered by rapidly rising health risks (Eichenbaum et al., 2020).[1]

1 Historical evidence supports the view that the negative economic effects of pandemics are large even when governments do not intervene with severe lockdowns as in the current COVID-19 case (Barro et al., 2020; Carillo and Jappelli, 2020; Jordà et al., 2020).

Table 1
**Descriptive statistics**

| | Mean | | | Median | | | Standard deviation | | | Minimum | | | Maximum | | | Countries |
|---|---|---|---|---|---|---|---|---|---|---|---|---|---|---|---|---|
| | Q1 | Q2 | Q3 | Q1 | Q2 | Q3 | Q1 | Q2 | Q3 | Q1 | Q2 | Q3 | Q1 | Q2 | Q3 | |
| Economic indicator | | | | | | | | | | | | | | | | |
| Growth rate (%) | -0.57 | -11.01 | -3.90 | -0.23 | -10.50 | -3.97 | 2.73 | 5.60 | 3.87 | -6.80 | -23.47 | -11.65 | 4.72 | 3.20 | 8.13 | 42 |
| COVID-19 | | | | | | | | | | | | | | | | |
| Fatality | 1.82 | 15.34 | 7.48 | 0.24 | 5.93 | 2.27 | 4.03 | 18.47 | 12.42 | 0.00 | 0.09 | 0.00 | 19.12 | 73.71 | 45.52 | 42 |
| Stringency | 20.06 | 68.12 | 53.16 | 18.76 | 70.00 | 52.72 | 8.35 | 10.27 | 15.22 | 6.85 | 38.51 | 24.48 | 58.98 | 87.23 | 83.66 | 42 |
| Controls | | | | | | | | | | | | | | | | |
| Trade | | 92.07 | | | 79.55 | | | 47.14 | | | 27.56 | | | 211.51 | | 42 |
| Tourism | | 7.76 | | | 5.37 | | | 6.03 | | | 1.52 | | | 26.38 | | 42 |
| GDP per capita (ln) | | 10.35 | | | 10.42 | | | 0.50 | | | 8.85 | | | 11.17 | | 42 |
| Trend growth (%) | | 2.87 | | | 2.48 | | | 1.91 | | | -1.25 | | | 9.88 | | 42 |
| Instruments | | | | | | | | | | | | | | | | |
| Speed | | 66.38 | | | 70.00 | | | 11.99 | | | 22.00 | | | 84.00 | | 42 |
| Life expectancy | | 79.21 | | | 81.16 | | | 4.27 | | | 63.86 | | | 84.21 | | 42 |
| Population (ln) | | 9.99 | | | 9.79 | | | 1.77 | | | 5.82 | | | 14.17 | | 42 |

Notes: All logarithmic values are scaled by ln(x+1). Growth rate is drawn from quarterly national account data provided by the OECD representing growth rates in percent of real GDP, change over the same quarter, previous year. Trend growth is drawn from OECD representing the mean average GDP growth rate over the period 2014-2019. COVID-19 variables are taken from the Oxford stringency index (Hale et al. 2020) database and calculated as quarter means. Controls are drawn from the World Bank Database representing 2018 values. Trade represents the sum of exports and imports divided by GDP. Tourism is measured by tourism receipts in total exports. Speed refers to the number of days it took from 1 January 2020 for governments to enact mandatory measures representing a stringency index level of 20 and above (i.e. Speed takes the value 76 if the stringency index stood at a level of 20 for the first time on 17 March 2020). Life expectancy is the mean value of total years at birth. Population (ln) is the total population (in thousands) and ln(x+1), e.g. ln(1,427,648+1)=14.17 for China for 2018.

Source: Authors' own elaboration.

The debate reemerged in the second wave. Again, some countries responded swiftly to the new rise in infections while other countries (e.g. Germany) opted for a staggered approach given that the measures enforced by the government in spring had been criticised as unreasonably harsh in economic terms considering the low fatality rates recorded at that time (Winkler, 2020).

The debate has been controversial and ongoing because even with increasingly available data, endogeneity challenges loom large. For example, the negative economic effects of mandatory measures will likely be relatively larger when enacted at rather low fatality rates, but governments are inclined to impose stricter measures for mandatory social distancing when fatality rates rise. Finally, the degree of voluntary social distancing is likely driven by country characteristics such as social cohesion and life expectancy. Against this background, we make use of three econometric approaches. First, we run a simple Ordinary Least Squares (OLS) analysis for each quarter with data available. Second, we account for endogeneity by instrumenting the stringency of government measures taken and the fatality rate. Third, we run panel fixed effects regressions controlling for time-invariant country characteristics and time fixed effects.

Our analysis builds on studies showing that risk aversion rises when people are confronted with COVID-19 cases in the region in which they live (Huynh, 2020; Maloney and Taskin, 2020). Thus, they provide direct evidence for the view that health risks, captured by the COVID-19 fatality rate, lead to voluntary social distancing and hence lower levels of economic activity. As a result, the economic impact of government-imposed measures is likely to be smaller than commonly assumed as the counterfactual is not the smooth pre-pandemic environment, but an economy operating under substantial voluntary social distancing. Goolsbee and Syverson (2020), Bartik et al. (2020), Gapen et al. (2020) and the latest World Economic Outlook (IMF, 2020) provide evidence for this view. By contrast, Dreger and Gros (2020) find that the stringency of government measures is the variable to look at when explaining developments in economic activity in EU member states between February and August 2020, while the negative economic effects of a rising fatality rate plays a rather unimportant role. This paper analyses whether the

Figure 1

**Average stringency index and fatality in the first, second and third quarter of 2020**

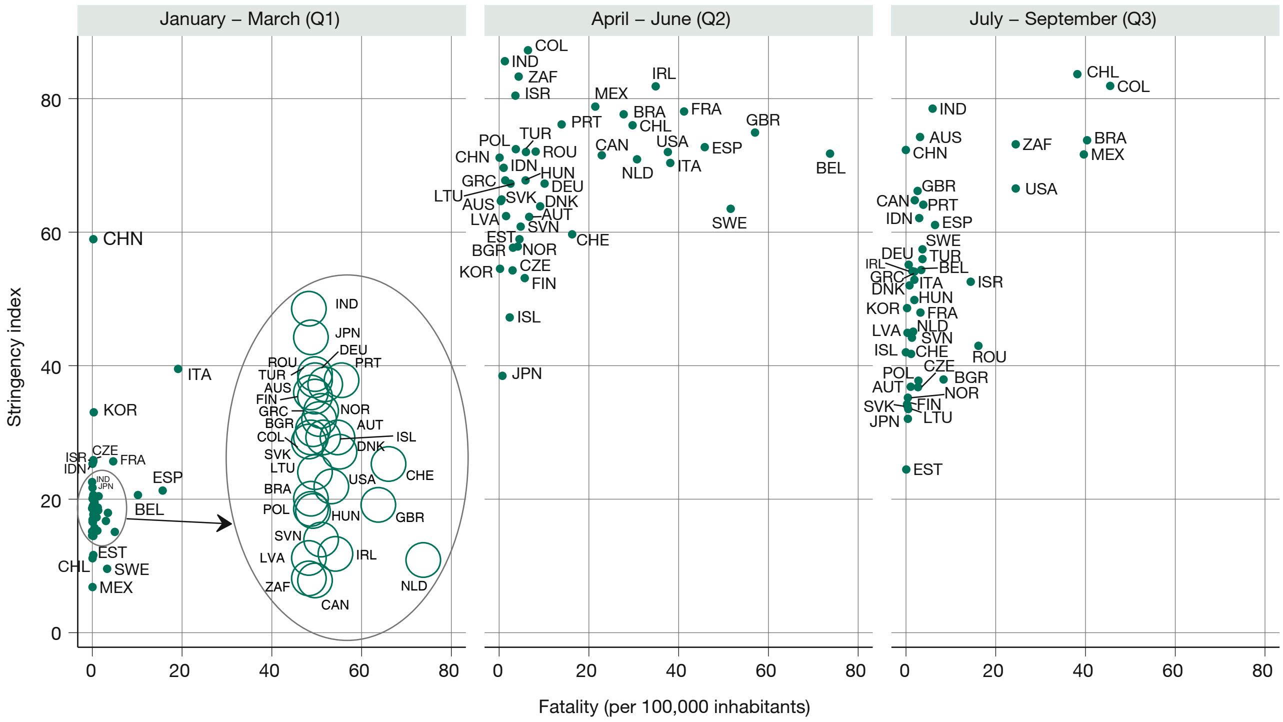

Sources: Hale et al. (2020) and authors' calculations.

fatality rate, i.e. the number of reported deaths related to COVID-19 (per 100,000 inhabitants), serving as a proxy for the severity of health risks triggering voluntary social distancing,[2] has a significantly negative effect on GDP developments in 42 countries for the first three quarters of 2020 while also accounting for lockdown severity, i.e. the degree of mandatory social distancing imposed by the authorities, captured by the Stringency index compiled by Oxford University (Hale et al., 2020).[3]

Results indicate that changes in the stringency of government measures dominate in-country GDP developments over time while the fatality rate plays an important role in explaining cross-country growth differences for each quarter. Moreover, social distancing abroad has a significantly negative effect on growth as countries with a larger exposure to tourism record a deeper fall in growth rates. We conclude from this that tighter government measures have a negative impact on economic activity but by keeping fatality rates low they might also support economic activity. Thus from an economic perspective, lockdowns might represent a second-best policy approach as they limit the economic damage associated with high fatality rates. Of course, more evidence is needed to reach firm conclusions as our analysis is based on evidence from three quarters only.

## Data and methodology

Our sample consists of 42 countries, including almost all of the OECD countries. Quarterly GDP growth, i.e. the change in real GDP over the same quarter in the previous year, serves as the dependent variable. Descriptive statistics (Table 1) show that countries record an average GDP growth rate of -0.6% in the first quarter before entering a deep recession with an average growth rate of -11% in the second quarter. Finally, the third quarter sees a recovery as the decline in GDP compared to the same quarter in the previous year drops to -3.9%. The striking differences between the three quarters also become visible when plotting stringency indices and fatality rates (Figure 1). In the first quarter, fatality rates are still low and

2 We opt for the fatality rate rather than the rate of infections as the latter is allegedly subject to larger cross-country differences unrelated to health risks triggered by COVID-19, such as different testing and reporting policies, than the former.

3 The countries are Australia, Austria, Belgium, Bulgaria, Brazil, Canada, Switzerland, Chile, China, Columbia, the Czech Republic, Germany, Denmark, Spain, Estonia, Finland, France, the United Kingdom, Greece, Hungary, Indonesia, India, Ireland, Iceland, Israel, Italy, Japan, South Korea, Lithuania, Latvia, Mexico, the Netherlands, Norway, Poland, Portugal, Romania, the Slovak Republic, Slovenia, Sweden, Turkey, the United States and South Africa.

governments of most countries enact rather mild measures to contain the pandemic. By contrast, in the second quarter, when many countries record fatality rates above 20, only two countries (Japan and Iceland) impose measures with a stringency level below 50.[4] In the third quarter, the standard deviation of government imposed measures rises substantially as governments choose different COVID-19 response strategies, while most countries report rather low fatality rates.

The stringency of measures and the fatality rate affect domestic economic activity by mandatory and voluntary social distancing at home. However, given the high degree of integration, domestic activity is likely to respond to mandatory and voluntary social distancing abroad as well. This is most obvious for the tourism industry when non-residents are unable to reach their destinations either due to travel bans imposed by foreign governments or because they voluntarily cancel their trips when confronted with rising health risks (Gössling et al., 2020; IMF, 2020). Thus, we account for the share of tourism receipts in total exports in 2018 in order to capture a country's vulnerability to mandatory and voluntary social distancing abroad. For similar reasons, we also control for trade openness, measured by the sum of exports and imports divided by GDP in 2018. Finally, we follow Lane and Milesi-Ferretti (2011) and control for GDP per capita as well as the average GDP growth rate between 2014 and 2019.

We start by running robust OLS regressions as well as robust instrumental variable (IV) regressions for each quarter under observation, i.e. we estimate:

$$\Delta y_{i,2020} = \alpha + \beta_1 * COVID_i + \beta_2 * SP_i + \beta_3 * Z_i + \varepsilon_i, \quad (1)$$

where $y_i$ is the quarterly GDP growth rate of country *i* in either the first, second or third quarter of 2020. $COVID_i$ are our main variables of interest, i.e. the stringency index and fatality rate. $SP_i$ represents vulnerabilities of countries to COVID-19 spillovers from abroad via tourism and trade, and $Z_i$ represents our general controls. In the IV regressions we instrument both COVID-19 variables by:

- The number of days starting from 1 January 2020 it took governments to respond to the pandemic in the form of mandatory restrictions defined as a stringency index level of 20.[5] For example, in Germany the stringency level reached 20 for the first time on 29 February, representing the 60th day of the year, while in the UK the threshold was hit for the first time on 17 March, i.e. the variable takes the value 76. Our choice is motivated by the hypothesis that lockdown severity and fatality rates likely reflect the speed with which governments responded to the outbreak of the virus (König and Winkler, 2020a).

- The life expectancy at birth as reported in 2018. COVID-19 mortality rates increase substantially with age, making countries with higher life expectancy more vulnerable to the pandemic. Thus, countries with a higher life expectancy are likely to experience higher fatality rates and more stringent government measures.

- Country size, measured by the natural logarithm of population size. Larger countries are likely to be more heterogeneous in terms of attitude and hence exhibit less social cohesion (Anckar, 1999; Gerring and Veenendaal, 2020). This might make it more challenging to keep the pandemic under control by relying mainly on voluntary social distancing. Thus, larger countries likely need tighter government imposed measures and/or (have to) accept higher fatality rates.

We continue by employing robust fixed effects panel regressions, i.e. we run the equation (1) as a panel regression replacing all time invariant country characteristics by country fixed effects and including time fixed effects. The observation period runs from 2014 Q1 to 2020 Q3, with stringency and fatality set to zero for all quarters until 2020 Q1.[6] In an extension, we also include lagged COVID-19 variables testing for longer-run effects of lockdowns and fatality.

## Results

Results of our OLS regressions (Table 2) indicate that cross-country differences in the stringency index drive cross-country growth differences in the first, but not in the second quarter, while the opposite holds for the fatality rate. In the third quarter, both COVID-19 variables are insignificant. Moreover, tourism exposure and GDP per capita account significantly for cross-country differences in GDP growth in the second and third quarter of 2020. The economic significance of tourism exposure can be illustrated for Greece, the country with the highest tourism exposure in 2018 within our sample. Second quarter GDP growth rate was -15% in Greece, of which about eight percentage points (26.38 * -0.3) are explained by the negative impact of mandatory and voluntary social distancing

4 Sebhatu et al. (2020) analyse the homogeneity of the response across countries.

5 The benchmark is close to the mean of the stringency index observed in the first quarter of 2020 for the country sample that our analysis is based upon.

6 The observation period begins in 2014 Q1 in order to exclude the effects of the global financial and euro crises on GDP developments. In total, we capture 27 quarters and time fixed effects until 2020 Q3.

Table 2
**OLS regressions**

| Dependent variable: Growth rate in % | (1) Q1 | (2) Q2 | (3) Q3 |
|---|---|---|---|
| Stringency | -0.17** [0.07] | -0.12 [0.09] | -0.04 [0.04] |
| Fatality | -0.10 [0.08] | -0.12** [0.05] | -0.03 [0.06] |
| Trade | -0.01 [0.01] | -0.02 [0.02] | -0.01 [0.01] |
| Tourism | -0.05 [0.08] | -0.30*** [0.10] | -0.19** [0.09] |
| GDP per capita (ln) | -0.30 [0.63] | 4.40* [2.20] | 2.37* [1.18] |
| Trend growth | 0.72*** [0.16] | 1.01 [0.62] | 1.09*** [0.37] |
| Constant | 5.70 [6.92] | -45.15* [26.09] | -26.85** [12.98] |
| Adjusted $R^2$ | 0.37 | 0.41 | 0.40 |
| Countries | 42 | 42 | 42 |
| F-Statistic | 8.64 | 6.28 | 4.41 |

Notes: OLS estimations. Robust standard errors. * denotes significance at 10%, ** significance at 5% and *** significance at 1%. For further notes see Table 1.

Source: Authors' estimation.

abroad via tourism exposure. By contrast, trade openness does not significantly explain cross-country differences in GDP growth. Finally, in line with expectations, richer countries fared better compared to countries with a lower per capita income in the second and third quarter, while trend growth fails to be significant at the peak of the pandemic's first wave in the second quarter only.

Overall, the OLS results suggest that in the beginning of the pandemic, when fatality rates are still rather low, differences in the stringency of the government's response matter, while in the second quarter, when basically all countries adopt tough measures, cross-country growth differences are driven by cross-country differences in the fatality rate. By contrast, the third quarter bears some resemblance to a 'normal' quarter in pre-pandemic times as both COVID-19 variables are insignificant and trend growth regains the status of a significant driver of cross-country growth differences it recorded in the first quarter.

We continue by instrumenting stringency and fatality with the variables referred to in the previous section (Table 3). Results of the first stage regressions show that a slower government response (Speed) is associated with a significantly higher stringency index (Q2) and fatality rate (Q3). Population size significantly explains the stringency index in the third and the fatality rate in the first and second quarter. Finally, life expectancy, while insignificant in explaining the stringency index, is positively associated with the fatality rate in the second quarter, when the first wave peaked. Results for the second stage regression show that the instrumented fatality rate is significant in the second and third quarter, while the instrumented stringency index is significant in the second quarter only. Moreover, tourism exposure is significant in the second and third quarter, while trade openness again fails to be significant. Overall, the IV regressions suggest that the fatality rate has been a more important driver of growth differences than the stringency index.[7] In particular third quarter results are consistent with the view that not only the stringency of government-imposed measures but also the COVID-19 death toll have had a negative impact on growth as the instrumented fatality rate remains significant in a quarter when government-imposed restrictions were reduced. In addition, tourism exposure, i.e. the vulnerability of countries to mandatory and social distancing abroad, is again identified as an important factor when explaining growth differences across countries in the second and third quarter.

Finally, we run panel fixed effects regressions (Table 4) in order to focus on the time dimension of the pandemic's impact on growth. Results show that changes in the stringency index over time drive GDP growth when accounting for both COVID-19 variables (column 3). Moreover, time fixed effects for the first and third quarter of 2020 are insignificant when accounting for the stringency index (columns 1 and 3). Thus, the divergence in growth in those quarters from the long-term average is significantly explained by changes in the stringency index only. However, the time fixed effect for the second quarter, while substantially smaller than in the specification with the fatality rate only (column 2), is significantly negative. Given that Q2 marks the peak of the first wave of the pandemic, this result indicates that changes in the stringency index over time do not completely account for the severe drop in GDP growth recorded in late spring and early summer 2020. Against this backdrop, we follow Barro et al. (2020) and expand the panel regression analysis by including the lag (i.e. one quarter) of the stringency index and the fatality rate as additional independent variables (columns 4-6).

Results show that the lagged fatality rate is insignificant in all specifications. By contrast, stronger government-imposed measures in time t=0 are associated with lower growth in the quarter they are enacted (Stringency $\beta$: -0.14***, column 6) but with higher growth (Stringency

7 This conclusion is reinforced by IV regressions (available on request) in which we account for only one of the COVID-19 variables for each quarter.

Table 3
**Instrumental variable regressions**

| Dependent variable: Growth rate in % | (1) Q1 | (2) Q2 | (3) Q3 | (4) Q1 | (5) Q2 | (6 Q3 |
|---|---|---|---|---|---|---|
| | Second stage (Stringency instrumented) | | | Second stage (Fatality instrumented) | | |
| Stringency | -0.13<br>[0.08] | -0.59**<br>[0.23] | -0.07<br>[0.10] | -0.14<br>[0.09] | 0.14<br>[0.16] | 0.03<br>[0.05] |
| Fatality | -0.14*<br>[0.08] | 0.03<br>[0.07] | -0.01<br>[0.09] | -0.31<br>[0.24] | -0.36**<br>[0.14] | -0.18*<br>[0.09] |
| Trade | -0.01<br>[0.01] | -0.03<br>[0.02] | -0.02<br>[0.01] | -0.01<br>[0.01] | -0.02<br>[0.02] | -0.01<br>[0.01] |
| Tourism | -0.03<br>[0.07] | -0.29*<br>[0.16] | -0.19**<br>[0.08] | -0.03<br>[0.08] | -0.40***<br>[0.09] | -0.22***<br>[0.08] |
| GDP per capita (ln) | -0.30<br>[0.56] | -0.84<br>[2.55] | 2.30**<br>[1.04] | -0.03<br>[0.73] | 9.15**<br>[3.90] | 1.23<br>[1.43] |
| Trend growth | 0.64***<br>[0.19] | 1.62***<br>[0.63] | 1.17***<br>[0.36] | 0.59***<br>[0.21] | 0.42<br>[0.70] | 0.77<br>[0.50] |
| Constant | 4.57<br>[6.07] | 37.31<br>[37.07] | -24.00**<br>[12.15] | 2.47<br>[8.32] | -106.12**<br>[46.92] | -16.64<br>[15.71] |
| Countries | 42 | 42 | 42 | 42 | 42 | 42 |
| Adjusted $R^2$ | 0.35 | -0.02 | 0.38 | 0.29 | 0.00 | 0.26 |
| F-Statistic | 6.73 | 6.76 | 3.86 | 2.01 | 2.82 | 5.04 |
| Sargan (p-Value) | 0.25 | 0.64 | 0.09 | 0.42 | 0.32 | 0.43 |
| Wooldridge (p-Value) | 0.22 | 0.05 | 0.68 | 0.34 | 0.04 | 0.13 |
| | First stage (Dep. var.: Stringency) | | | First stage (Dep. var.: Fatality) | | |
| Speed | -0.52***<br>[0.13] | 0.32***<br>[0.08] | 0.26<br>[0.16] | 0.12<br>[0.10] | 0.29<br>[0.22] | 0.36**<br>[0.17] |
| Life Expectancy | 0.20<br>[0.29] | -0.06<br>[0.61] | 0.49<br>[0.76] | 0.31<br>[0.20] | 1.42**<br>[0.69] | 0.19<br>[0.79] |
| Population (ln) | -0.21<br>[0.59] | 1.95<br>[1.51] | 4.41***<br>[1.37] | 1.10**<br>[0.46] | 4.89**<br>[2.32] | -1.15<br>[1.67] |
| Countries | 42 | 42 | 42 | 42 | 42 | 42 |
| Adjusted $R^2$ | 0.66 | 0.48 | 0.58 | 0.18 | 0.40 | 0.53 |

Notes: See Table 1.

Source: Authors' estimation.

(lag) β: 0.11*, column 6) in the following quarter (t=1). Thus, the immediate negative effects on economic activity triggered by a strong government response to rising health risks are partly reversed in the next period as countries with a higher stringency index in the previous period record higher growth in the current period. Finally, it has to be noted that the time fixed effect for the second quarter of 2020 remains significantly negative. Thus, even the specification with lagged COVID-19 variables does not fully capture the depth of the recession in that quarter.

As a robustness check, we rerun all specifications with the stringency index and the fatality rate in natural log form (ln (x+1)). Results, available from the authors on request, point to a somewhat more pronounced role of the fatality rate. For the OLS and IV regressions, the fatality rate is again found to be the more powerful COVID-19 variable while in the panel without lags, both COVID-19 variables are now found to be significant. By contrast, none of the lagged COVID-19 variables are significant when employing them in a logarithmic form.

## Conclusions

The question of whether and to what extent the COVID-19-induced recession is linked to the stringency of government-imposed measures enforcing social distancing and whether and to what extent the recession is also caused by voluntary distancing related to rising health risks has been intensively debated among economists, policymakers and the public at large. Exploiting evidence on GDP growth in 42 countries over the first, second and third quarter of 2020, we find that changes in lockdown stringency are the more important driver of GDP devel-

Table 4
**Panel fixed effects regressions**

| Dependent variable: Growth rate in % | (1) | (2) | (3) | (4) | (5) | (6) |
|---|---|---|---|---|---|---|
| Stringency | -0.13*** | - | -0.11*** | -0.16*** | - | -0.14*** |
| | [0.03] | - | [0.04] | [0.03] | - | [0.04] |
| Fatality | - | -0.09** | -0.05 | - | -0.09** | -0.05 |
| | - | [0.03] | [0.04] | - | [0.03] | [0.04] |
| Fatality (lag) | - | - | - | -0.00 | -0.01 | 0.00 |
| | - | - | - | [0.03] | [0.03] | [0.03] |
| Stringency (lag) | - | - | - | 0.11* | 0.05 | 0.11* |
| | - | - | - | [0.06] | [0.07] | [0.06] |
| 2020 Q1 | -0.03 | -2.57*** | -0.39 | 0.53 | -2.57*** | 0.18 |
| | [0.78] | [0.40] | [0.91] | [0.83] | [0.40] | [0.96] |
| 2020 Q2 | -3.99* | -11.81*** | -4.76** | -4.38* | -12.70*** | -5.09* |
| | [2.03] | [0.94] | [2.31] | [2.19] | [1.16] | [2.53] |
| 2020 Q3 | 1.10 | -5.40*** | 0.28 | -5.16 | -8.50** | -5.86 |
| | [1.77] | [0.52] | [2.05] | [3.63] | [4.18] | [3.69] |
| Constant | 2.16*** | 2.16*** | 2.16*** | 2.16*** | 2.16*** | 2.16*** |
| | [0.20] | [0.20] | [0.20] | [0.20] | [0.20] | [0.20] |
| Model | | Time fixed effects | | | Time fixed effects | |
| Countries | 42 | 42 | 42 | 42 | 42 | 42 |
| $R^2$ (within) | 0.66 | 0.65 | 0.67 | 0.67 | 0.66 | 0.67 |
| $R^2$ (overall) | 0.50 | 0.50 | 0.51 | 0.51 | 0.51 | 0.51 |
| $R^2$ (between) | 0.00 | 0.10 | 0.00 | 0.00 | 0.15 | 0.02 |
| Rho (inter. cor.) | 0.48 | 0.46 | 0.47 | 0.48 | 0.46 | 0.48 |
| F-Statistic | 60.82 | 80.41 | 75.39 | 68.02 | 73.82 | 71.16 |

Notes: Fixed effects model. Robust standard errors. * denotes significance at 10%, ** significance at 5% and *** significance at 1%. Observation period for Growth rate begins in 2014 Q1. Time fixed effects represent in total 27 quarters until 2020 Q3, Stringency and Fatality is equal to zero until 2020 Q1. For further notes see Table 1.

Source: Authors' estimation.

opments over time. When including lagged variables, two effects can be distinguished: a negative one as more restrictive measures lead to lower GDP growth in the same quarter, and a positive, catching-up effect associated with stringency developments lagged by one period. OLS and IV regressions for each quarter suggest that voluntary social distancing reflecting differences in health risks expressed by the fatality rate also play a substantial role in explaining cross-country differences in GDP growth. Moreover, these regressions show that country vulnerabilities to mandatory and voluntary social distancing conducted abroad, proxied by tourism exposure, matter.

We interpret our results as providing broad support for the policy approach taken by most countries in the coronavirus pandemic when managing perceived and real trade-offs between health and economic risks. On the one hand, results confirm the conventional view that from an economic perspective, all efforts should be undertaken to avoid hard lockdowns as any rise in lockdown intensity has severely negative effects on economic activity. At the same time, our cross-country results also suggest that high fatality rates are associated with strongly negative growth effects. Thus, our results also support those voices arguing that tight lockdowns – despite their negative effect on growth – might still serve as a useful economic policy instrument if they succeed in reducing health risks as economic activity is severely hampered by high fatality rates. Having said this, we want to conclude by noting that our results are based on evidence for three quarters only. Thus, they will need to be reexamined when new data becomes available.
